# Biosynthesis and functions of sulfur modifications in tRNA

**DOI:** 10.3389/fgene.2014.00067

**Published:** 2014-04-02

**Authors:** Naoki Shigi

**Affiliations:** Biomedical Research Institute, National Institute of Advanced Industrial Science and TechnologyTokyo, Japan

**Keywords:** post-transcriptional modification, post-translational modification, sulfur, tRNA, ubiquitin

## Abstract

Sulfur is an essential element for a variety of cellular constituents in all living organisms. In tRNA molecules, there are many sulfur-containing nucleosides, such as the derivatives of 2-thiouridine (s^2^U), 4-thiouridine (s^4^U), 2-thiocytidine (s^2^C), and 2-methylthioadenosine (ms^2^A). Earlier studies established the functions of these modifications for accurate and efficient translation, including proper recognition of the codons in mRNA or stabilization of tRNA structure. In many cases, the biosynthesis of these sulfur modifications starts with cysteine desulfurases, which catalyze the generation of persulfide (an activated form of sulfur) from cysteine. Many sulfur-carrier proteins are responsible for delivering this activated sulfur to each biosynthesis pathway. Finally, specific “modification enzymes” activate target tRNAs and then incorporate sulfur atoms. Intriguingly, the biosynthesis of 2-thiouridine in all domains of life is functionally and evolutionarily related to the ubiquitin-like post-translational modification system of cellular proteins in eukaryotes. This review summarizes the recent characterization of the biosynthesis of sulfur modifications in tRNA and the novel roles of this modification in cellular functions in various model organisms, with a special emphasis on 2-thiouridine derivatives. Each biosynthesis pathway of sulfur-containing molecules is mutually modulated via sulfur trafficking, and 2-thiouridine and codon usage bias have been proposed to control the translation of specific genes.

## INTRODUCTION

A characteristic structural and functional feature of RNA is post-transcriptional modification. More than 100 forms of naturally occurring chemical modification have been reported to date^1^,^2^ (Cantara et al., 2011; Machnicka et al., 2013). The roles of modified nucleosides in tRNA are important and wide-ranging, and include critical roles in biogenesis, structural stability, codon recognition, maintenance of reading frame, and identification elements for the translation machinery (Björk, 1995; Curran, 1998).

**FIGURE 1:**
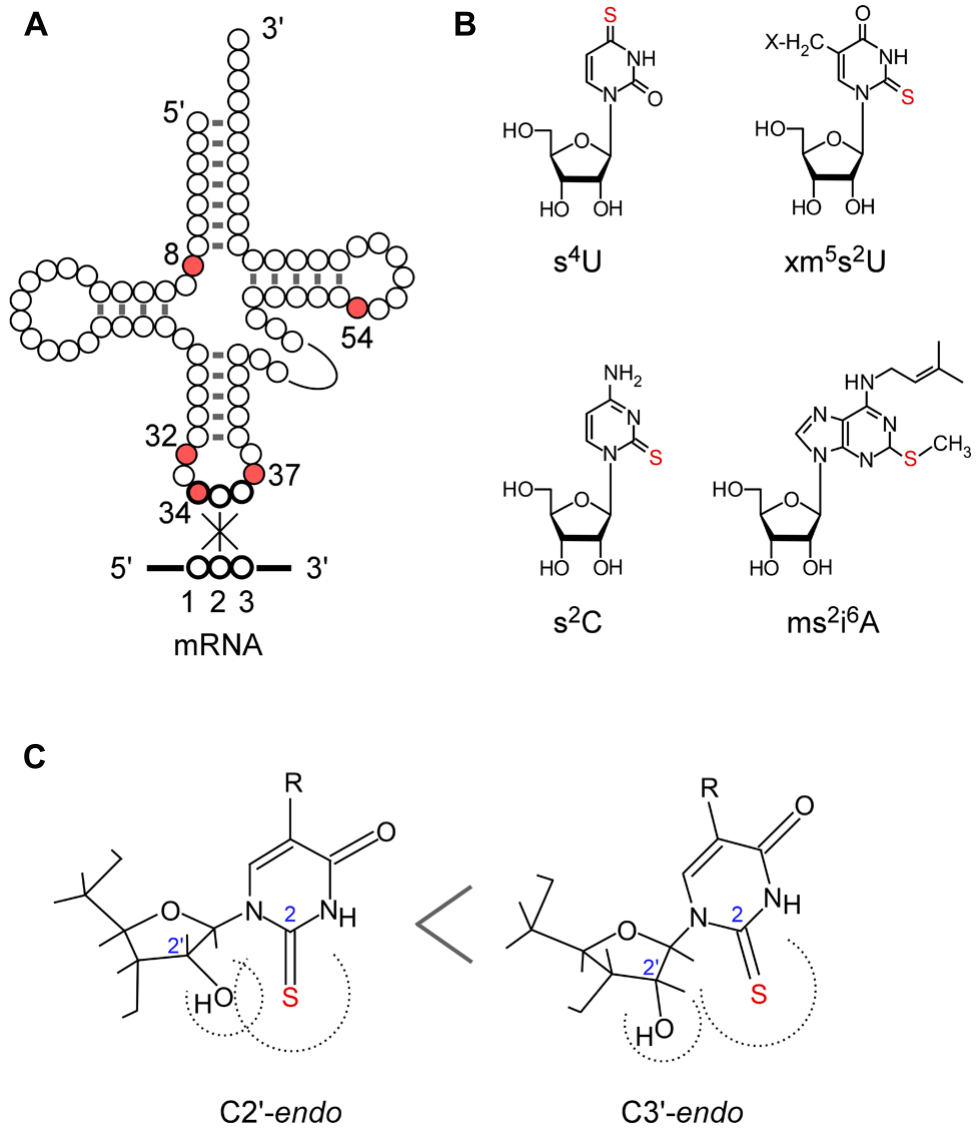
**Sulfur-containing tRNA modifications**. **(A)** Secondary structure of tRNA and positions of thiolated nucleosides in tRNA. **(B)** Chemical structure of thiolated nucleosides in *E. coli*: s^4^U, 4-thiouridine; s^2^C, 2-thiocytidine; xm^5^s^2^U, 5-methyl-2-thiouridine derivatives; ms^2^i^6^A, 2-methylthio-*N*^6^-isopentenyladenosine. **(C)** Conformation of the xm^5^s^2^U: C3’ -endo form is preferred because of the steric hindrance of the 2-thio and 2’ -OH groups.

**FIGURE 2:**
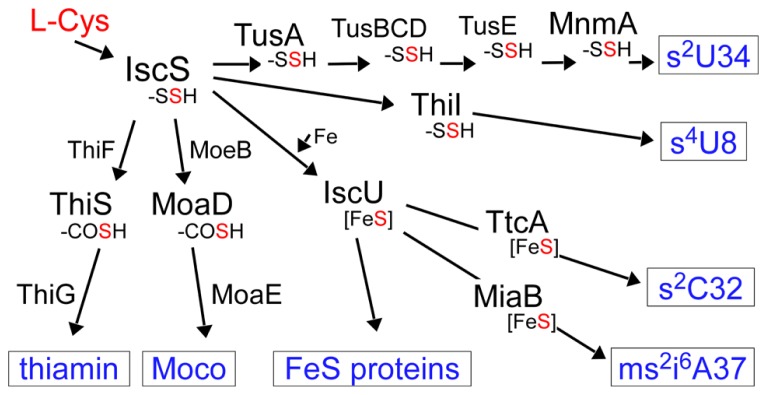
**Sulfur flow in the biosynthesis of various sulfur-containing molecules in *E. coli***. The sulfur atom in cysteine is first activated by IscS as a persulfide. The persulfide sulfur on IscS is delivered to acceptor proteins in each pathway. -SSH: persulfide, -COSH: thiocarboxylate, Fe–S: iron–sulfur cluster, Moco: molybdenum cofactor.

The biosynthesis and functions of thionucleosides have been elucidated mainly by using *Escherichia coli*, *Salmonella enterica* serovar Typhimurium, and *Saccharomyces cerevisiae* as model organisms. *E. coli* tRNAs contain five thionucleosides, 4-thiouridine (s^4^U) at position 8, 2-thiocytidine (s^2^C) at position 32, 5-methylaminomethyl-2-thiouridine (mnm^5^s^2^U) or 5-carboxymethylaminomethyl-2-thiouridine (cmnm^5^s^2^U) at position 34, and 2-methylthio-*N*^6^-isopentenyladenosine (ms^2^i^6^A) at position 37 (**Figure 1**). The biosynthesis of these thionucleosides can be divided into two major groups depending on the involvement of iron–sulfur (Fe–S) cluster biosynthesis. The thiouridines s^4^U8 and (c)mnm^5^s^2^U34 are synthesized independently of Fe–S cluster formation, while s^2^C32 and ms^2^i^6^A37 synthesis is dependent upon Fe–S cluster formation, which suggest that Fe–S-containing proteins are present in the latter biosynthesis pathways (**Figure 2**; Lauhon et al., 2004; Leipuviene et al., 2004).

The first step of mobilization of sulfur in both pathways starts with the activation of the sulfur atom of cysteine by an enzyme, cysteine desulfurase, IscS (**Figure 2**). IscS forms an enzyme-bound persulfide (IscS–SSH) using pyridoxal-5’-phospate (PLP) as a cofactor and this activated sulfur is transferred to the next acceptor protein in each pathway. For the biosynthesis of s^4^U8 and (c)mnm^5^s^2^U34, the sulfur atom is transferred to specific sulfur-carrier proteins or a “modification enzyme” (Palenchar et al., 2000; Ikeuchi et al., 2006). The modification enzymes bind and activate target tRNA and catalyze the final step of sulfur transfer to tRNA. For the biosynthesis of s^2^C32 and ms^2^i^6^A37 (Fe–S cluster dependent pathway), the persulfide generated by IscS is transferred to the “scaffold” protein IscU, on which the Fe–S cluster is synthesized, and the Fe–S cluster is then incorporated into the modification enzymes for ms^2^i^6^A37 (and maybe also for s^2^C32; Pierrel et al., 2002, 2003, 2004; Jäger et al., 2004). In ms^2^i^6^A synthesis, it was reported that the sulfur atom in the Fe–S cluster is not the sulfur donor (Forouhar et al., 2013); therefore, the ultimate sulfur donor *in vivo* remains to be determined.

The sulfur atom activated by IscS is also used in molybdenum cofactor (Moco) and thiamin biosynthesis (**Figure 2**). These are sulfur-containing cofactors whose biosynthesis also includes unique sulfur-carrier proteins. Moco is incorporated into the active sites of many molybdoenzymes, including nitrate reductase, sulfite oxidase, and xanthine dehydrogenase (Schindelin et al., 2001). Moco contains a molybdenum atom and a pterin named molybdpterin (MPT). In MPT biosynthesis, two sulfur atoms are incorporated into precursor Z using a protein-thiocarboxylate as a sulfur donor. Thiamin is an essential cofactor for enzymes involved in carbohydrate and branched-chain amino acid metabolism and is synthesized from thiazole and pyrimidine moieties (Settembre et al., 2003). The sulfur atom of the thiazole ring is added in most bacteria by a system similar to the Moco biosynthetic machinery.

In *S. cerevisiae*, there are two thiouridines in tRNA, 5-methoxycarbonylmethyl-2-thiouridine (mcm^5^s^2^U34) in cytosolic tRNAs and 5-carboxymethylaminomethyl-2-thiouridine (cmnm^5^s^2^U34) in mitochondrial tRNAs. The biosynthesis pathway of 2-thiouridine in cytosolic tRNA is Fe–S cluster dependent, while the mitochondrial pathway is independent of Fe–S cluster formation (Umeda et al., 2005; Nakai et al., 2007). The biosynthesis of s^2^U in cytosolic tRNA in the eukaryote utilizes a protein-thiocarboxylate as intermediate sulfur donor. This pathway is functionally and evolutionarily related to the ubiquitin-like post-translational modification system of cellular proteins in eukaryotes and a similar biosynthesis pathway in archaea was reported (Humbard et al., 2010; Miranda et al., 2011).

In some thermophiles, 5-methyl-2-thiouridine (m^5^s^2^U) [also called 2-thioribothymidine (s^2^T)] occurs at position 54 in the T-loop (**Figure 1**). Intriguingly, the biosynthesis pathway (Shigi et al., 2008) is similar to that of cytosolic s^2^U34 in eukaryotes, and ubiquitin-like post-translational modification of cellular proteins has recently been discovered also in the bacteria domain (Shigi, 2012).

In this review, I summarize recent advances with respect to the characterization of the biosynthesis mechanisms of sulfur modifications in tRNA, with special reference to 2-thiouridine derivatives. Up to the time of writing, two major pathways for the biosynthesis of 2-thiouridine have been reported. These pathways differ in terms of the types of modification enzyme and the ultimate sulfur donor (**Table 1**). The novel roles of 2-thiouridine in cellular functions have been revealed by new techniques including genome-wide analyses in some model organisms. Interestingly, each biosynthesis pathway to sulfur-containing molecules has been suggested to be mutually modulated via sulfur trafficking and translational control of specific genes by 2-thiouridine derivatives in tRNAs.

**Table 1:** Two pathways of 2-thiouridine biosynthesis.

Strain	Modified base	Position	Function	Modification enzyme	Activated sulfur species
Bacteria	xm^5^s^2^U	34	Decoding	MnmA type	Protein-persulfide
Eukaryote (mitochondria)	xm^5^s^2^U	34	Decoding	MnmA type	Protein-persulfide
Eukaryote (cytosol)	xm^5^s^2^U	34	Decoding	Ncs6/TtuA type	Protein-thiocarboxylate, Protein-persulfide
Bacteria (thermophile)	m^5^s^2^U (s^2^T)	54	Thermal stabilization	Ncs6/TtuA type	Protein-thiocarboxylate, Protein-persulfide

## FUNCTIONAL PROPERTIES OF 2-THIOURIDINE BASED ON ITS STRUCTURE

The 2-thiouridine modification at position 34 and 54 plays critical roles in protein synthesis. Position 34 (the wobble base) of tRNAs for Glu, Gln, and Lys are universally modified to 5-methyl-2-thiouridine derivatives (xm^5^s^2^U; **Figure 1**): 5-methylaminomethyl-2-thiouridine (mnm^5^s^2^U) and 5-carboxymethylaminomethyl-2-thiouridine (cmnm^5^s^2^U) in bacterial tRNAs, 5-methoxycarbonylmethyl-2-thiouridine (mcm^5^s^2^U) in eukaryotic cytosolic tRNAs, cmnm^5^s^2^U in yeast mitochondrial tRNA, and 5-taurinomethyl-2-thiouridine (τm^5^s^2^U) in mammalian mitochondrial tRNAs (Suzuki, 2005).

The conformation of xm^5^s^2^U preferentially takes the C3’-*endo* form of ribose puckering, because of the steric effect of the bulky 2-thiocarbonyl group toward the 2’-hydroxyl group (**Figure 1C**; Yokoyama et al., 1985; Agris et al., 1992). The xm^5^s^2^U34 base pairs preferentially with purines and prevents misreading of near cognate codons ending in pyrimidines (Agris et al., 1973; Yokoyama et al., 1985; Murphy et al., 2004; Durant et al., 2005; Johansson et al., 2008) and frame shifting (Urbonavicius et al., 2001; Atkins and Björk, 2009; Isak and Ryden-Aulin, 2009; Jäger et al., 2013). The 2-thio group of xm^5^s^2^U34 is required for efficient codon recognition on the ribosome (Ashraf et al., 1999; Vendeix et al., 2012; Rodriguez-Hernandez et al., 2013). In addition, the 2-thio group of cmnm^5^s^2^U34 in tRNA^Glu^acts as the identity element for specific recognition by glutaminyl-tRNA synthetase (Sylvers et al., 1993; Rodriguez-Hernandez et al., 2013). In human, a defect in mitochondrial translation is induced by the lack of xm^5^s^2^U34 modification in mutant mitochondrial tRNA^Lys^ from patients with myoclonus epilepsy with ragged-red fibers (MERRF; Yasukawa et al., 2000, 2001).

The 2-thio modification of m^5^s^2^U (s^2^T) at position 54 in the T-loop also plays an important role in protein synthesis in high temperature environments. In thermophilic organisms such as *Thermus thermophilus* and *Pyrococcus furiosus*, almost all tRNA species are modified to m^5^U54 and m^5^s^2^U54 (Watanabe et al., 1974; Kowalak et al., 1994). The m^5^s^2^U54 is also found in the hyperthermophilic bacterium *Aquifex aeolicus* (Awai et al., 2009). The 2-thiolation content of m^5^U54 increases with cultivation temperature (Watanabe et al., 1976; Kowalak et al., 1994). As deletion strains of *T. thermophilus* lacking the 2-thio group of the m^5^s^2^U54 modification show a temperature sensitive phenotype, this modification is suggested to be required for survival of the thermophile at high temperature (Shigi et al., 2006a). In the L-shaped tRNA structure, m^5^s^2^U54 is buried inside the tertiary core and forms a reverse Hoogsteen base pair with m^1^A58 and also stacking with G53 and ψ55. In addition, ψ55 and C56 form tertiary base pairs with G18 and G19 in the D-loop, respectively. The rigid conformation of m^5^s^2^U54 stabilizes the A-form helix of the D-loop–T-loop interaction, contributing to the thermostability of tRNAs in the thermophile (Watanabe et al., 1974; Horie et al., 1985).

## MnmA PATHWAY FOR 2-THIOURIDINE SYNTHESIS IN BACTERIA AND EUKARYOTE ORGANELLES

**FIGURE 3:**
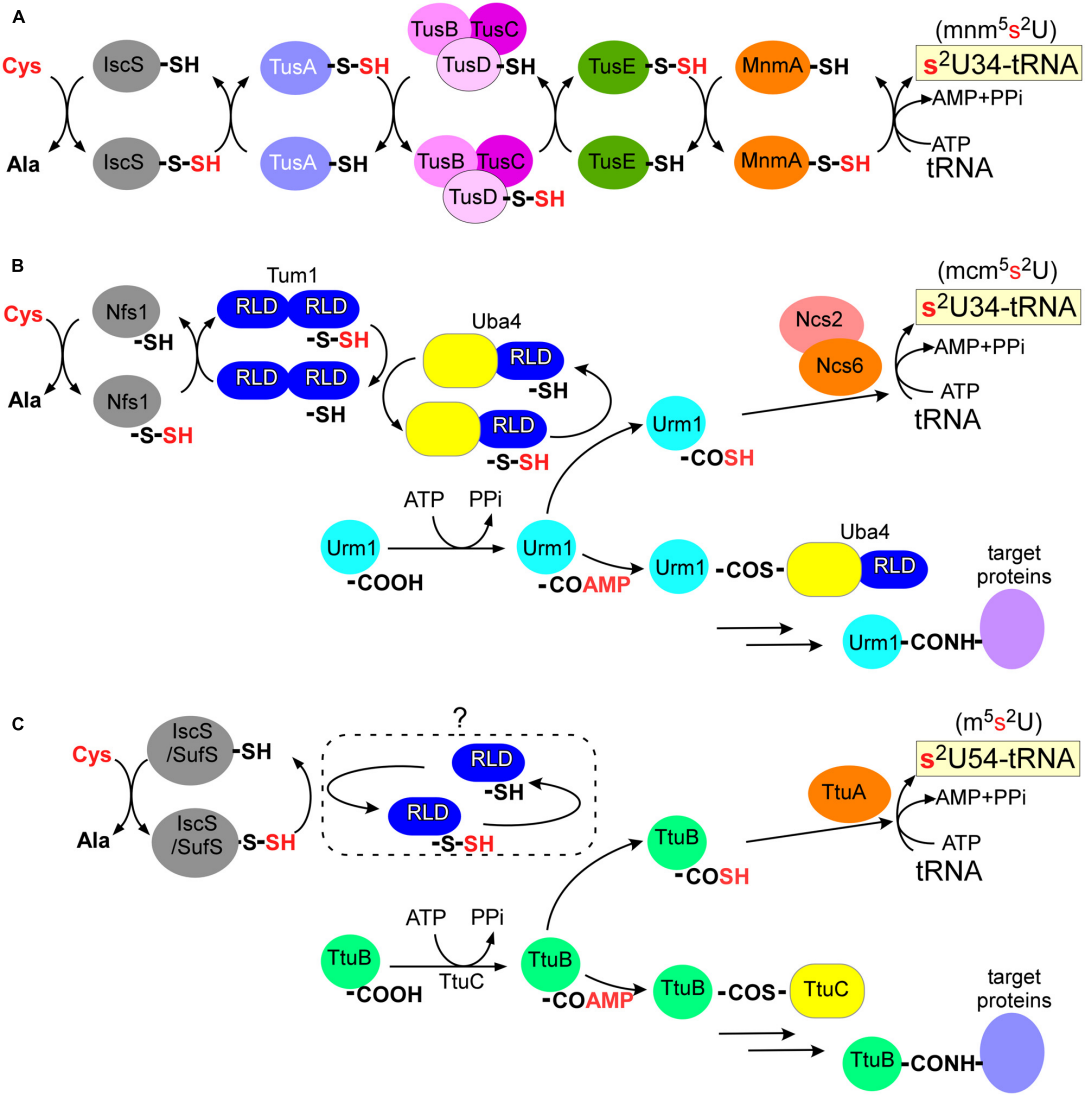
**Biosynthesis pathway of 2-thiouridine derivatives**. **(A)** In *E. coli*, Tus proteins relay the persulfide sulfur of IscS to a modification enzyme, MnmA. **(B)** In *S. cerevisiae*, Urm1 acts both as sulfur carrier and protein modifier. A thioester conjugate or an acyldisulfide conjugate (not shown) of Urm1 and Uba4 were proposed in urmylation pathway. RLD: rhodanese-like domain. **(C)** In *T. thermophilus*, TtuB also acts both as sulfur carrier and protein modifier. An unidentified RLD protein might be involved in sulfur transfer.

In *E. coli*, seven proteins are responsible for 2-thiolation of 5-methylaminomethyl-2-thiouridine (mnm^5^s^2^U) or 5-carboxymethylaminomethyl-2-thiouridine (cmnm^5^s^2^U) in the wobble base of tRNA^Glu^_UUC_, tRNA^Gln^_UUG_, and tRNA^Lys^_UUU_: a cysteine desulfurase (IscS), a modification enzyme (MnmA), and three persulfide carriers (TusA, TusBCD complex, and TusE; **Figure 3A**; Kambampati and Lauhon, 2003; Ikeuchi et al., 2006; Numata et al., 2006a). The sulfur atom of L-cysteine is first activated by IscS cysteine desulfurase to form an enzyme-bound persulfide. The small sulfur-carrier proteins TusA, TusBCD, and TusE relay this sulfur atom via their active site cysteine residues to MnmA. Tus proteins stimulate sulfur transfer from IscS to the catalytic cysteine of MnmA (Cys199). MnmA is an N-type ATP-pyrophosphatase that possesses the characteristic PP-motif (Bork and Koonin, 1994) and two conserved cysteine residues (Cys102 and Cys199). The reaction mechanism was well documented in a biochemical study based on the crystal structure of the MnmA-tRNA complex (Numata et al., 2006b). MnmA binds the anticodon arm and D-stem regions of tRNA and activates the C2-position of the uracil ring at position 34 as an acyl-adenylated intermediate (tRNA-OAMP). This is then followed by nucleophilic attack by the persulfide sulfur of MnmA-Cys199-SSH, which results in the completion of 2-thiouridine formation.

Genomic analysis of bacteria revealed that IscS, TusA, and MnmA are mostly conserved, whereas TusBCD and TusE are not found in many organisms (Kotera et al., 2010). This implies that a variation in the sulfur-transfer pathways from IscS to MnmA may exist. In eukaryotic mitochondria, NifS and Mtu1 (homologs of IscS and MnmA, respectively) are responsible for 2-thiolation of cmnm^5^s^2^U in yeast and 5-taurinomethyl-2-thiouridine (τ m^5^s^2^U) in mammals (Umeda et al., 2005). In eukaryotic mitochondria, the intermediate sulfur carriers remained to be identified.

## Ncs6/Urm1 PATHWAY FOR 2-THIOURIDINE SYNTHESIS IN THE CYTOSOL OF EUKARYOTES

The Ncs6/Urm1 pathway is responsible for the 2-thiolation of 5-methoxycarbonylmethyl-2-thiouridine (mcm^5^s^2^U) in the wobble base of tRNA^Glu^_UUC_, tRNA^Gln^_UUG_, and tRNA^Lys^_UUU_ in the cytosol of eukaryotes (*S. cerevisiae*, *Schizosaccharomyces pombe*, *Caenorhabditis elegans*, *Homo sapiens*; Esberg et al., 2006; Björk et al., 2007; Dewez et al., 2008; Huang et al., 2008; Nakai et al., 2008; Schlieker et al., 2008; Schmitz et al., 2008; Leidel et al., 2009; Noma et al., 2009). A similar pathway was reported subsequently in plants (Leiber et al., 2010; Nakai et al., 2012). With the exception of the first step catalyzed by cysteine desulfurase Nfs1, the eukaryotic pathway is quite different from the MnmA pathway in bacteria described above. The function of Nfs1 is to donate the sulfur to the Fe–S cluster and 2-thiouridine. Formation of 2-thiouridine is dependent on Fe–S cluster biosynthesis (ISC) and cytosolic Fe–S cluster assembly (CIA) machineries in yeast (Nakai et al., 2007). This suggests that the Ncs6/Urm1 pathway depends on Fe–S protein(s), although at the time of writing it remains to be determined which protein(s) possess Fe–S cluster(s).

The Ncs6/Urm1 pathway is composed of at least six proteins including a cysteine desulfurase (Nfs1), a modification enzyme complex (Ncs6/Ncs2), two sulfur carriers (Urm1 and Tum1), and an activation enzyme for Urm1 (Uba4; **Figure 3B**). The gene names here are those of *S. cerevisiae*, and homologs of Ncs6/Ncs2 and Uba4 in humans are designated ATPBD3/CTU2 and MOCS3 (molybdenum cofactor synthesis 3), respectively. Tum1 and Uba4 contain rhodanese-like domains (RLDs) bearing conserved cysteine residues. Rhodanese is a widespread sulfur-carrier enzyme that catalyzes sulfur-transfer reactions in various metabolic pathways (Bordo and Bork, 2002). The conserved cysteine residues of RLDs in Tum1 and Uba4 are critical for 2-thiouridine formation *in vivo*. Tum1 probably directs sulfur flow to 2-thiouridine formation (Noma et al., 2009). The persulfide of Nfs1 is transferred to the RLD of Uba4 mainly via the RLD of Tum1.

**FIGURE 4:**
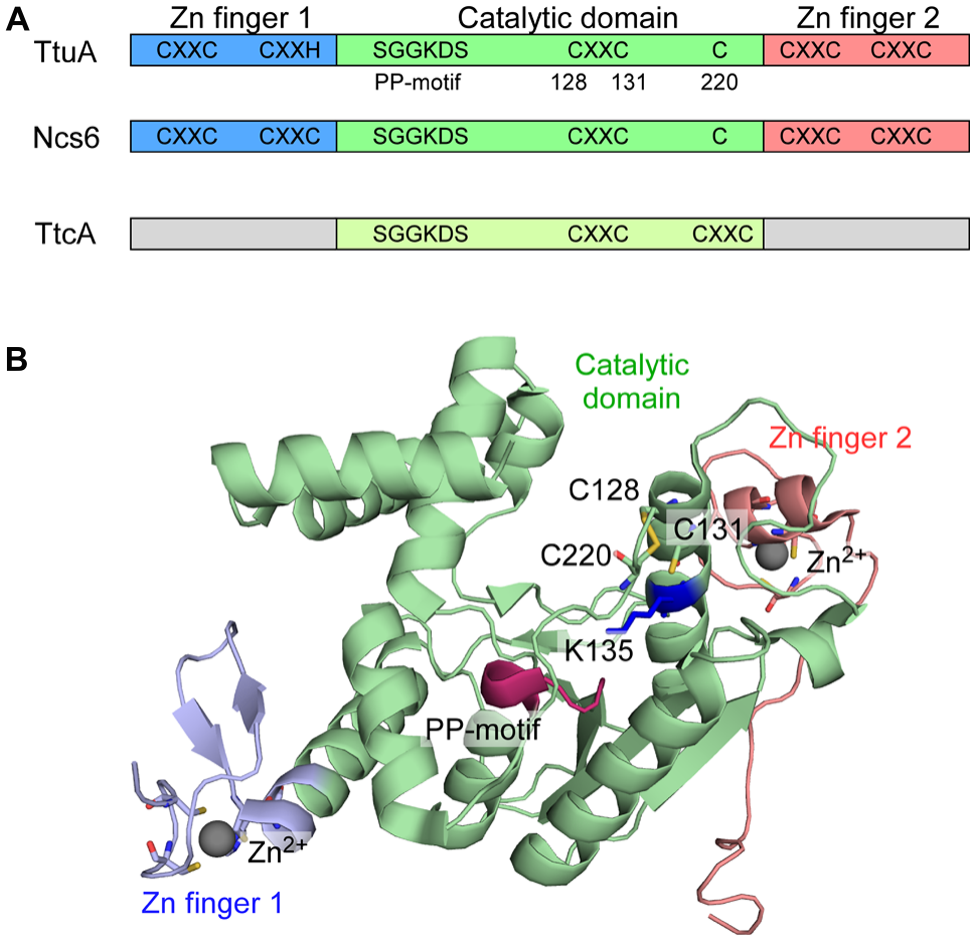
**TtuA and Ncs6 proteins in 2-thiouridine biosynthesis**. **(A)** Schematic representation of TtcA family proteins. TtuA and Ncs6 belong to group II of the TtcA family. TtuA is composed of two Zn finger domains and a catalytic domain. TtcA is responsible for the formation of 2-thiocytidine34. **(B)** Structure of *P. horikoshii* TtuA. The three domains are colored the same as in **(A)**. The PP-motif is shown in red. Three Cys residues that is important for enzyme activity is shown with a stick representation. The target K135 for TtuB conjugation (K137 in *T. thermophilus*) is shown in a blue stick model.

Urm1 is a ubiquitin-related modifier and Uba4 is an E1-like Urm1-activating enzyme involved in protein urmylation (see the following; Furukawa et al., 2000). The carboxy-terminus of Urm1 is first activated as an acyl-adenylate intermediate (Urm1-COAMP) and then thiocarboxylated (Urm1-COSH) by a persulfide from the RLD of Uba4 (**Figure 3B**; Schlieker et al., 2008; Schmitz et al., 2008; Leidel et al., 2009; Noma et al., 2009). The activated thiocarboxylate may be utilized in subsequent reactions for 2-thiouridine formation, which is mediated by a heterodimer complex, Ncs6/Ncs2 (Dewez et al., 2008; Noma et al., 2009). Ncs6 has the PP-motif and many CXXC motifs (**Figure 4A**; see TtuA/TtuB Pathway for 2-Thiouridine Synthesis in Thermophile tRNAs). Thus, 2-thiolation of mcm^5^s^2^U shares a pathway and chemical reactions with protein urmylation. Intriguingly, eukaryotic 2-thiouridine formation employs a thiocarboxylated intermediate as the active form of the sulfur atom, which is a mechanism distinct from bacterial sulfur-relay based on persulfide chemistry.

## POST-TRANSLATIONAL MODIFICATION OF CELLULAR PROTEINS BY Urm1 IN EUKARYOTES

Ubiquitin (Ub) and ubiquitin-like proteins (Ubls) are post-translational protein modifiers with important roles in proteolysis and the regulation of diverse processes in eukaryotes (Hochstrasser, 2009). The breakdown of the Ub/Ubl system is often associated with the development of various diseases. In the first step of conjugation to target proteins, the conserved C-terminal glycine of Ub/Ubl is acyl-adenylated by an activating enzyme (E1) and covalently linked to a cysteine residue of E1 to form an Ub/Ubl-E1 thioester intermediate. The activated Ub/Ubl is next transferred to a conjugating enzyme (E2). Finally, Ub/Ubl is attached to a lysine residue in the target protein by a ligase (E3; Hochstrasser, 2009).

Proteins homologous to eukaryotic Ub/Ubl and E1s exist in almost all members of bacteria and archaea (Iyer et al., 2006; Burroughs et al., 2009, 2012). Earlier works established that these bacterial proteins function in the biosynthesis of sulfur compounds such as molybdenum cofactor and thiamin (Kessler, 2006). Bacterial Ubls (MoaD and ThiS) are adenylated by cognate E1 homologs (MoeB and ThiF), subsequently bind activated sulfur via their C-termini to form thiocarboxylates, and finally act as sulfur donors (**Figure 2**; Pitterle and Rajagopalan, 1993; Taylor et al., 1998; Lauhon and Kambampati, 2000; Leimkühler et al., 2001; Zhang et al., 2010). These findings imply an evolutionary link between the eukaryotic Ub/Ubl system and the bacterial sulfur-transfer reaction (Iyer et al., 2006; Hochstrasser, 2009). Urm1 is an ubiquitin-related modifier and Uba4 is an E1-like enzyme involved in protein urmylation in eukaryotes (Furukawa et al., 2000; **Figure 3B**). A thioester conjugate (Furukawa et al., 2000) or an acyldisulfide conjugate (Van der Veen et al., 2011) of Urm1 and Uba4 were proposed in this process. As Urm1 also functions as a sulfur donor for 2-thiouridine synthesis (see the preceding) and has close sequence and structural homology with bacterial Ubls (Xu et al., 2006), Urm1 is thought to be the most ancient Ubl possessing dual functions of protein modifier and sulfur carrier. The E2 and E3 enzymes for urmylation have not been identified at the time of writing.

Several targets of urmylation have been identified upon cell exposure to an oxidant (Van der Veen et al., 2011), although earlier reports only identified a peroxiredoxin Ahp1 (Goehring et al., 2003a, b). Among them, a modification enzyme complex ATPBD3/CTU2 and an E1-like MOCS3, both of which are required for 2-thiouridine biosynthesis, have been identified. The target residues in these proteins have not been identified and the roles of urmylation of these proteins are unknown at the time of writing; however, regulation of the activities of these enzymes would be possible.

## TtuA/TtuB PATHWAY FOR 2-THIOURIDINE SYNTHESIS IN THERMOPHILE tRNAs

Prior work from our group identified the TtuA/TtuB pathway for the biosynthesis of thiouridine (m^5^s^2^U) at position 54 in tRNAs from a thermophilic bacterium *T. thermophilus*. The TtuA/TtuB pathway includes cysteine desulfurases (IscS or SufS), a modification enzyme (TtuA), a small ubiquitin-like sulfur carrier (TtuB), and an activation enzyme for TtuB (TtuC; **Figure 3C**; Shigi et al., 2006a, b, 2008). Similar to the eukaryotic Ncs6/Urm1 pathway described above, the C-terminal Gly of TtuB is acyl-adenylated (TtuB-COAMP) by TtuC and is then thiocarboxylated (TtuB-COSH) by cysteine desulfurases (IscS or SufS). The sulfur atom of the thiocarboxylated TtuB is transferred to tRNA by TtuA. This step also requires ATP as a cofactor and TtuA possesses the PP-motif, suggesting that TtuA may activate the target uridine as an acyl-adenylate. The sulfur-transfer activity in the *in vitro* system requires the addition of cell-free extract and the activity was low, suggesting that there may still be additional factors required for TtuA-mediated sulfur transfer to tRNA.

TtuA and eukaryotic Ncs6 are homologous to each other, and belong to group II of the TtcA family, whose members are characterized by five conserved CXXC(H) motifs and the PP-motif (**Figure 4A**; Bork and Koonin, 1994; Jäger et al., 2004; Björk et al., 2007). TtcA, which catalyzes 2-thiocytidine (s^2^C) synthesis (Jäger et al., 2004), has only two CXXC motifs and the PP-motif, and therefore belongs to group I of the TtcA family. The PP-motif is used for ATP binding to adenylate the target nucleotide, and is widely distributed among ATP pyrophosphatases, including modification enzymes MnmA (see the preceding; Numata et al., 2006b), ThiI for 4-thiouridine (s^4^U) synthesis (Mueller and Palenchar, 1999; Palenchar et al., 2000), and TilS for tRNA^Ile2^ lysidine synthesis (Ikeuchi et al., 2005).

We determined the crystal structure of the TtuA homolog (PH0300) of the archeaon *P. horikoshii* (Nakagawa et al., 2013). The *P. horikoshii* genome has two TtuA/Ncs6-like ORFs: one (PH0300) seems to be an ortholog of *T. thermophilus* TtuA; and the other (PH1680) seems to be an ortholog of eukaryotic Ncs6, based on their sequence homology to TtuA and Ncs6. The *P. horikoshii* TtuA forms a homodimer, and each subunit contains a catalytic domain and unique N- and C-terminal zinc fingers (**Figure 4B**). The N-terminal zinc finger is made up of the first and second CXXC/H motifs, where the zinc atom is coordinated by three Cys residues and one His residue. The C-terminal zinc finger is made up of the fourth and fifth CXXC motifs, where the zinc atom is coordinated by four Cys residues.

Interestingly, the catalytic domain of TtuA has much higher structural similarity to that of another tRNA modification enzyme, TilS (tRNA^Ile2^ lysidine synthetase), than to the other type of tRNA 2-thiolation enzyme, MnmA (**Figure 4**). However, three Cys residues (128, 131, 220 in PhTtuA) are clustered in the putative catalytic site, which are absent in TilS. Cys128 and Cys131 are in the third CXXC motif and Cys220 is also conserved. By *in vivo* mutational analysis of TtuA in *T. thermophilus* (Nakagawa et al., 2013), it became apparent that the three conserved cysteine residues and the putative ATP-binding residues are important for TtuA activity, implying a key role for these Cys residues in sulfur transfer from TtuB-COSH to tRNA. A positively charged surface that includes the catalytic site and the two zinc fingers is likely to provide the tRNA binding site. TtuA recognizes the T-loop (Shigi et al., 2002) and Ncs6/Ncs2 is predicted to recognize the anticodon arm. The recognition mechanisms of the different target sites on tRNA require clarification.

## POST-TRANSLATIONAL MODIFICATION OF CELLULAR PROTEINS BY TtuB IN A BACTERIUM *T. thermophilus*

Homology modeling suggests that TtuB possesses a Ub/β-grasp fold and TtuC has significant sequence homology with the adenylation domain of eukaryotic E1s (Shigi et al., 2008). These findings suggest that Ub/Ubl homologous conjugation systems also exist in bacteria. A series of *in vivo* analyses in *T. thermophilus* revealed that TtuB is covalently attached to target proteins most likely via its C-terminal glycine (Shigi, 2012). TtuC is required for conjugate formation, and TtuC and TtuA are targets for TtuB conjugation. Mass spectrometric analysis combined with *in vivo* mutational analysis revealed that lysine residues (K137/K226/K229) in TtuA are covalently modified by the C-terminal carboxylate of TtuB. K137 in *T. thermophilus* TtuA is situated just after the third CXXC motif. In the crystal structure of *P. horikoshii* TtuA, K137 (K135 in PhTtuA) is situated close to the catalytic center of this enzyme family (**Figure 4B**; Nakagawa et al., 2013). K137 in TtuA is conserved in related bacteria and archaea, such as *Aquifex*, *Pyrococcus*, *Thermococcus*, and *Metanocaldococcus*. On the other hand, this position is occupied by a conserved arginine in eukaryotic Ncs6. K226 and K229 in *T. thermophilus* TtuA are situated just after Cys222 (Cys220 in PhTtuA), although the regions near the two lysine residues were disordered in the PhTtuA structure. However, K226 and K229 are only conserved in *T. thermophilus* and a few other species, possibly implying species-specific functions of the conjugation. Intriguingly, a deletion mutant of a JAMM [JAB1/MPN/Mov34 metalloenzyme (Ambroggio et al., 2004)] ubiquitin isopeptidase homolog in *T. thermophilus* showed aberrant TtuB-conjugates of TtuC and TtuA, and a ~50% decrease in the amount of thiouridine in tRNA (Shigi, 2012). These results support the hypothesis that thiouridine synthesis is regulated by TtuB conjugation.

## THE CASE IN ARCHAEA

Although the precise chemical structure of the archaeal counterpart remains unknown at the time of writing, the existence of modified uridines at the wobble position in tRNA^Lys^ and tRNA^Glu^from *Haloferax volcanii* has been reported (Gupta, 1984). The existence of 2-thiouridine in tRNA^Lys^ from *H. valcanii* was suggested by APM-electrophoresis (Miranda et al., 2011), a method that can detect sulfur modifications in RNAs (Igloi, 1988). Because the homologs of eukaryotic Ncs6 are widely distributed in archaeal genomes, these proteins may be involved in this modification (Kotera et al., 2010). Genetic analysis in *H. valcanii* shows that SAMP2 (small archaeal modifier protein 2) and E1-like protein UbaA are required for thiouridine formation in this organism (Miranda et al., 2011). These results indirectly suggest that SAMP2-COSH is formed and used as a sulfur donor for thiouridine formation in archaea, as observed previously in eukaryotes and bacteria. SAMPs are the first example of a ubiquitin-like protein modifier identified other than from a eukaryote (Humbard et al., 2010) and extensive studies show that the archaeal protein modification system resembles that of eukaryotes in many aspects (Van der Veen et al., 2011; Hepowit et al., 2012; Miranda et al., 2014). SAMP2 covalently conjugates too many target proteins including UbaA (a Uba4 homolog), HVO_0580 (a Ncs6 homolog), and HVO_0025 (a Tum1 homolog) (Humbard et al., 2010), implying that the SAMP2 modification also regulates the thiolation machinery.

4-Thiouridine (s^4^U), is a modified nucleotide of tRNA that is conserved from bacteria to archaea, where it functions as a sensor for near-UV irradiation (Favre et al., 1969; Carre et al., 1974; Ryals et al., 1982). Sulfur transfer in the biosynthesis of s^4^U has been extensively studied in bacteria (Kambampati and Lauhon, 2000; Mueller et al., 2001; Leipuviene et al., 2004). The persulfide of IscS is transferred to the RLD of ThiI, a PP-motif-containing modification enzyme. Recently, ThiI lacking an RLD domain has been characterized in methanogenic archaea (Liu et al., 2012). It has three cysteine residues (two of which come from a CXXC motif) in the putative catalytic site, and they are all required for persulfide intermediate formation. This may provide a hint about the catalytic mechanism of TtuA/Ncs6, because of the sequence and structural similarities between TtuA and ThiI (Waterman et al., 2006; Nakagawa et al., 2013).

## BIOSYNTHESIS NETWORK OF SULFUR-CONTAINING MOLECULES

The mobilization of sulfur in biosynthesis pathways of sulfur-containing compounds starts with the activation of the sulfur atom of cysteine by the cysteine desulfurase IscS. IscS forms an enzyme-bound persulfide and this activated sulfur is transferred to the next acceptor protein in each pathway, such as TusA (2-thiouridine in tRNA), ThiI (4-thiouridine in tRNA), IscU (Fe–S cluster), ThiS (thiamin), and MoaD (molybdenum cofactor; **Figure 2**). It is conceivable that each biosynthesis pathway of sulfur-containing molecules is mutually modulated via competition of sulfur trafficking. An interesting observation with respect this was made during a study of lambda phage infection in *E. coli* (Maynard et al., 2010, 2012). During viral infection, the normal amount of modified uridine in tRNA^Lys^_UUU_ grarantees a normal translation and frameshifting rate for production of the proper ratio of viral gpG and gpGT proteins (gpGT production needs programmed ribosomal frameshifting). Hypomodification of tRNA^Lys^_UUU_caused by deletion of Tus genes in the host cell leads to increased frameshifting in the translation of viral mRNA of G and T genes, which affects the ratio of viral gpG to gpGT. A lower gpG:gpGT ratio leads to decreased virion production. Another factor lowering infection is overexpression of IscU in the host cell. In this situation, higher sulfur flow from IscS to IscU conversely lowers the sulfur flow to Tus proteins, which leads to hypomodification of tRNA^Lys^_UUU_ and abnormal frameshifting, which finally affects the viral infection rate. The competitive binding of TusA and IscU to IscS has been analyzed in detail, based on the structures of the complexes IscS/TusA and IscS/IscU (Shi et al., 2010; Marinoni et al., 2012).

The sharing of a factor downstream of cysteine desulfurase also occurs in this sulfur-transfer network. TusA was originally identified as a sulfur carrier for 2-thiouridine synthesis (Ikeuchi et al., 2006); however, it was later reported to be involved in Moco synthesis in *E. coli* (Dahl et al., 2013; Kozmin et al., 2013). It has been proposed that the deletion of TusA leads to the overproduction of Fe–S clusters, which finally affects the expression of several genes (Dahl et al., 2013). A study of the link between the MnmA pathway and cellular redox state has recently been reported (Nakayashiki et al., 2013). By screening for mutants sensitive to hydroxyurea (HU) in *E. coli*, the authors identified mutations in all genes in the MnmA pathway (*iscS, mnmA,* and *tusA-E* in 2-thiouridine synthesis at the wobble position). These mutations resulted in a more reduced state, which may have led to a change in the activity of ribonucleotide reductase, an enzyme inhibited by HU. It is possible that a change in sulfur flow to each pathway in mutants of the MnmA pathway led to the reduced cellular state, although the precise mechanism underlying this phenomenon still remains unknown.

There is another interesting case. In *E. coli*, bacterial Ubls (MoaD and ThiS) are adenylated by cognate E1 homologs (MoeB and ThiF), subsequently bind activated sulfur at their C-termini to form thiocarboxylate, and work as sulfur donors for Moco and thiamin biosynthesis, respectively (**Figure 2**; Kessler, 2006). In the *T. thermophilus* genome, there is only one E1 homolog, TtuC. The *ttuC* mutant cannot synthesize 2-thio modification of m^5^s^2^U54; moreover, Moco and thiamin biosynthesis are also defective. TtuC and cysteine desulfurase can activate and thiocarboxylate TtuB, MoaD, and ThiS *in vitro*. Thus, TtuC is a common E1-like enzyme shared by the biosynthesis pathways of these three sulfur-containing compounds (Shigi et al., 2008). Similarly, in human, MOCS2A (a MoaD homolog) and URM1 are adenylated by an E1-like MOCS3 (Uba4 homolog; Chowdhury et al., 2012). In archaea, E1-like UbaA is involved in the biosynthesis of Moco and thiouridine (Miranda et al., 2011). Interestingly, these E1 homologs are also involved in the post-translational modification of cellular proteins in all domains of life (Furukawa et al., 2000; Miranda et al., 2011; Shigi, 2012).

## FUNCTION VIA TRANSLATIONAL CONTROL OF A SPECIFIC GROUP OF GENES IN EUKARYOTES

The inactivation of the genes in the Ncs6/Urm1 pathway results in a pleotropic phenotype that includes increased sensitivity to high temperature, oxidative stress, and rapamycin, a Tor-signaling inhibitor (Furukawa et al., 2000; Goehring et al., 2003a, b; Dewez et al., 2008). These phenotypes can be suppressed by overexpression of tRNA^Lys^, tRNA^Glu^, and tRNA^Gln^, which normally possess mcm^5^s^2^U34 (Leidel et al., 2009). The phenotypes are similar to those of mutants of the elongator complex (Esberg et al., 2006), which is essential for modification of the C5 position of U34. These observations provide evidence that the mcm^5^s^2^U modification in tRNAs affects global translation, resulting in a pleotropic phenotype.

In *S. cerevisiae*, pioneering work has addressed the function of tRNA modifications on gene expression. The mcm^5^ modification of the wobble base of specific tRNAs modulates the expression of a DNA damage response mRNA, whose cognate codons are unusually overrepresented (Begley et al., 2007). A similar observation has been made concerning telomeric gene silencing (Chen et al., 2011). Proteome analysis in *S. pombe* showed that the amount of a specific group of proteins, including those involved in cell division, was decreased in mutants defective in the mcm^5^s^2^U modification (Bauer and Hermand, 2012; Bauer et al., 2012). The genes coding for these proteins have skewed lysine codon usage, such that the AAA codon was overrepresented compared to the AAG codon. The mcm^5^s^2^U modification in tRNA^Lys^_UUU_ is necessary especially for the efficient translation of mRNAs enriched in the AAA codon. Among the genes affected by the mcm^5^s^2^U modification, a central regulator of mitosis and cytokinesis, Cdr2, was identified. The amount of Cdr2, a protein kinase, was recovered by overexpression of tRNA^Lys^_UUU_ in the mutant defective in mcm^5^s^2^U biosynthesis. In addition, after substituting AAG codons for all AAA codons in *cdr2* mRNA, the Cdr2 protein amount was no longer affected by the mcm^5^s^2^U modification. This study provides an interesting example of how translational control of a specific group of mRNAs can be affected by tRNA modifications and codon usage. The translation of specific genes in *S. pombe* and *S. cerevisiae* has also been reported by two other groups to be controlled by the s^2^U modification and codon usage (Fernandez-Vazquez et al., 2013; Rezgui et al., 2013).

In *S. cerevisiae*, 2-thiouridine formation in tRNA-Lys, -Glu, and -Gln is actively downregulated when methionine and cysteine are limiting, which leads to an overall reduction in translational capacity and reduced growth, because Glu, Gln, and especially Lys codons are overrepresented in the genes essential for translation and growth (Laxman et al., 2013). In this case, tRNA thiolation works as a key effector to maintain amino acid homeostasis.

## CONCLUSION AND FUTURE PERSPECTIVES

In the course of the characterization of biosynthesis pathways of sulfur-containing modifications, a number of sulfur-carrier proteins have been identified. The sulfur-carrier proteins may have evolved to deliver reactive sulfur atoms to specific targets and avoid non-specific transfer of activated sulfur atoms, which could inactivate other biomolecules. At the time of writing, it is still unclear whether or not the differences in the chemical properties of persulfide and thiocarboxylate result in different biological outcomes.

Each biosynthesis pathway of sulfur-containing molecules is mutually modulated by sulfur trafficking, and translational control of specific genes by 2-thiouridine and codon usage bias is now proposed in some model organisms. It would be interesting to determine whether similar mechanisms exist in higher eukaryotes.

Unexpectedly, the characterization of the biosynthesis of 2-thiouridine has revealed molecular fossils, namely, ancient ubiquitin-like molecules, including Urm1 in eukaryotes, TtuB in bacteria, and SAMP2 in archaea. These proteins have two functions; they function as sulfur carriers for 2-thiouridine synthesis and as protein modifiers. Therefore, these proteins may be evolutionarily intermediates between ancient sulfur-carrier proteins and protein modifiers. It is possible that an adenylated or thiocarboxylated intermediate, formed in the course of 2-thiouridine biosynthesis, was incidentally attached to adjacent proteins at some time in the past. By this post-translational modification, the activities of the attached proteins have probably changed. This was certainly the origin of the post-translational modification of proteins by these Ubls. The primitive function of these conjugates was probably self-regulation. Conjugates of Ubls and the modification enzymes Ncs6/Ncs2 and TtuA have already been detected, but the function of these post-translational modifications remains to be clarified. The resultant conjugates have become to be used as tags for the recognition and regulation of other proteins. By acquiring E2 and E3 enzymes, which can recognize target proteins precisely, the Ub system evolved considerably to become the sophisticated system it is today in eukaryotes.

## Conflict of Interest Statement

The author declares that the research was conducted in the absence of any commercial or financial relationships that could be construed as a potential conflict of interest.

## Footnotes

1 http://mods.rna.albany.edu/

2 http://modomics.genesilico.pl/
